# Convergence of plasmid-mediated Colistin and Tigecycline resistance in *Klebsiella pneumoniae*

**DOI:** 10.3389/fmicb.2023.1221428

**Published:** 2024-01-03

**Authors:** Yujie Zhao, Changrui Qian, Jianzhong Ye, Qingcao Li, Rongqing Zhao, Ling Qin, Qifeng Mao

**Affiliations:** ^1^Department of Clinical Laboratory, The Affiliated Li Huili Hospital, Ningbo University, Ningbo, China; ^2^Department of Clinical Laboratory, The First Affiliated Hospital of Wenzhou Medical University, Wenzhou, China; ^3^Department of Clinical Laboratory, Ningbo No. 2 Hospital, Ningbo, China

**Keywords:** colistin, MCR-1, tigecycline, tmexCD1-toprJ1, plasmid

## Abstract

**Objective:**

The co-occurrence of colistin and tigecycline resistance genes in *Klebsiella pneumoniae* poses a serious public health problem. This study aimed to characterize a *K. pneumoniae* strain, K82, co-harboring a colistin resistance gene (CoRG) and tigecycline resistance gene (TRG), and, importantly, investigate the genetic characteristics of the plasmid with CoRG or TRG in GenBank.

**Methods:**

*K. pneumoniae* strain K82 was subjected to antimicrobial susceptibility testing, conjugation assay, and whole-genome sequencing (WGS). In addition, comparative genomic analysis of CoRG or TRG-harboring plasmids from K82 and GenBank was conducted. *K. pneumoniae* strain K82 was resistant to all the tested antimicrobials including colistin and tigecycline, except for carbapenems.

**Results:**

WGS and bioinformatic analysis showed that K82 belonged to the ST656 sequence type and carried multiple drug resistance genes, including *mcr-1* and *tmexCD1-toprJ1*, which located on IncFIA/IncHI2/IncHI2A/IncN/IncR-type plasmid pK82-*mcr-1* and IncFIB/IncFII-type plasmid pK82-*tmexCD-toprJ*, respectively. The pK82-*mcr-1* plasmid was capable of conjugation. Analysis of the CoRG/TRG-harboring plasmid showed that *mcr-8* and *tmexCD1-toprJ1* were the most common CoRG and TRG of *Klebsiella* spp., respectively. These TRG/CoRG-harboring plasmids could be divided into two categories based on mash distance. Moreover, we found an IncFIB/IncHI1B-type plasmid, pSYCC1_tmex_287k, co-harboring *mcr-1* and *tmexCD1-toprJ1*. To the best of our knowledge, this is the first report on the co-occurrence of *mcr-1* and *tmexCD1-toprJ1* on a single plasmid.

**Conclusion:**

Our research expands the known diversity of CoRG and TRG-harboring plasmids in *K. pneumoniae*. Effective surveillance should be implemented to assess the prevalence of co-harboring CoRG and TRG in a single *K. pneumoniae* isolate or even a single plasmid.

## 1 Introduction

*Klebsiella pneumoniae* is one of the most important pathogens that can cause invasive hospital- and community-acquired infections, such as bacteremia, respiratory tract infections, and liver abscesses (Lee et al., 2017; David et al., 2019). With the extensive use of antibiotics, the rapid emergence of carbapenem-resistant hypervirulent *K. pneumoniae* (CR-hvKP) presents a severe challenge for clinical treatment (Shankar et al., 2018). In most cases, CR-hvKP is associated with an outbreak of infection in hospitals and high mortality rates (Yao et al., 2015). Therapeutic options for these species are mainly reliant on colistin and tigecycline, which are classified as critically important antimicrobials (He et al., 2019). Regrettably, the increasing use of colistin and tigecycline has inevitably resulted in the emergence of colistin and tigecycline-resistance isolates (Soliman et al., 2021).

Colistin resistance in *K. pneumonia* is commonly attributed to chromosomal mutations, including *mgrB*, *phoP*/*phoQ*, *pmrA*/*pmrB,* and *crrA*/*crrB* (Poirel et al., 2017). However, since the first discovery of *mcr-1* in *Escherichia coli* in 2015 (Liu et al., 2016), nine variants of this gene (*mcr-2* to *mcr-10*) have also been identified in *E. coli* and other *Enterobacteriaceae* strains (Gogry et al., 2021). In the persistent dissemination of colistin resistance, horizontal transfer of plasmid-borne *mcr* genes plays a significant role, which further worsens the severe situation (Wang et al., 2018). Until now, three plasmid-borne *mcr* genes, including *mcr-1*, *mcr-7*, and *mcr-8*, have been found in *K. pneumonia* (Xiaomin et al., 2020; Phetburom et al., 2021). In general, they led to the emergence of extensively drug-resistant *K. pneumoniae,* including colistin resistance along with other plasmid-mediated resistance genes, such as extended-spectrum beta-lactamase (ESBL) and carbapenem genes (Singh et al., 2021; Muraya et al., 2022).

Tigecycline resistance is a growing concern in gram-negative bacteria due to the emergence of plasmids containing mobile tigecycline-resistance genes, such as *tet*(X), *tet*(A), *tet*(K), and *tet*(M) variants, exacerbating transferable resistance between bacterial species (Linkevicius et al., 2016; Sun et al., 2019). New *tet*(X) variants have been identified from a variety of different bacterial species (Dong et al., 2022a; Lu et al., 2022, 2023). Among these *tet*(X) variants, the *tet*(X4) gene has been identified in a few studies in the *Klebsiella pneumoniae* strain, which poses a great threat to the clinical use of tigecycline (Li et al., 2022; Zhai et al., 2022). Recently, a novel resistance-nodulation-division (RND) efflux pump gene cluster, *tmexCD1-toprJ1,* encoded by plasmid was identified in *K. pneumoniae* isolates (Lv et al., 2020). Subsequently, its variants *tmexCD2-toprJ2*, *tmexCD3-toprJ3*, and *tmexCD4-toprJ4*, encoding tigecycline resistance, were revealed (Wang Y. et al., 2021; Gao et al., 2022). These highly transmissible resistance determinants are presenting a severe challenge for clinical management and treatment.

Worryingly, recent studies have found that *tmexCD1-toprJ1* can be co-transferred with other mobile resistance genes, such as *mcr-8.2*, *bla_NDM − 1,_* and *bla_kpc-2_* in *K. pneumoniae* (Liu et al., 2022, 2023). These plasmid-mediated resistance determinants are highly transmissible, presenting a severe challenge for clinical management. Furthermore, the emergence of colistin and tigecycline resistance determinants in the endemic *K. pneumoniae* clone constitutes a true public threat. In this study, we characterize an ST656 multidrug-resistant *K. pneumonia* isolate, harboring colistin resistance gene (CoRG) *mcr-1.1* and tigecycline resistance gene (TRG) *tmexCD1-toprJ1,* from the urine specimen of a bladder cancer patient. Moreover, we performed *in silico* typing and comparative analysis of CoRG or TRG-positive plasmids using the plasmids of *Klebsiella* species available in the NCBI RefSeq database. This study expands the diversity of known CoRG or TRG-carrying plasmids in *K. pneumoniae* strains and provides a basis for further prevention and control of the dissemination of such strains.

## 2 Materials and methods

### 2.1 Bacterial strains

The *K. pneumoniae* strain K82 was isolated from a urine sample from an 82-year-old man with a history of postoperative bladder cancer at Ningbo Medical Center, Li Huili Hospital in Zhejiang, China, in October 2016. The patient had a hospital-acquired urinary tract infection and was not treated with colistin or tigecycline. *E. coli* C600 (highly resistant rifampicin) were used as hosts for conjugal transfers. Strain K82 was initially identified by Vitek 2 Compact. Later, it was confirmed using matrix-assisted laser desorption ionization-time of flight mass spectrometry (MALDI-TOF MS; bioMérieux, France). It was stored at −80°C in Luria-Bertani (LB) broth medium (Oxoid, UK) with 30% glycerol for further use.

### 2.2 Antimicrobial susceptibility testing

*In vitro* susceptibility tests of ceftazidime, cefepime, aztreonam, imipenem, meropenem, piperacillin/tazobactam, cefoperazone/sulbactam, amikacin, tobramycin, trimethoprim-sulfamethoxazole, levofloxacin, and minocycline were performed using the Vitek 2 Compact in N335 susceptibility cards (bioMérieux, France). The minimum inhibitory concentrations (MICs) of colistin and tigecycline were determined using the microdilution broth method, and the results were determined in accordance with the 2023 Clinical and Laboratory Standards Association (CLSI) guidelines (Clinical and Laboratory Standards Institute [CLSI], 2023). *E. coli* ATCC 25922 was used as the quality control strain.

### 2.3 Conjugation experiments

Conjugation experiments were performed with rifampicin-resistant *E. coli* C600 and azide-resistant *E. coli* J53 as the recipient, and the transferability of *mcr-1* and *tmexCD1-toprJ1* genes was investigated using strain K82 as a donor. The donor and recipient strains were grown in 3 mL LB broth overnight at 37°C. Subsequently, 50 μL of donor strain culture was mixed with 500 μL of recipient strain culture (v:v = 1:10) and 4.5 mL of fresh LB broth (Luo et al., 2022). In addition, 100 μL of the mixture was applied onto a cellulose filter membrane (pore size, 0.22 μm) already placed on an LB agar plate. After incubation at 37°C for 16 h to 18 h, the filter membrane was taken out and vortexed in 1 mL of LB broth. The vortex mixtures were plated on LB agar plates containing 2000 mg/L rifampicin or 200 mg/L sodium azide, together with 1 mg/L colistin or 2 mg/L tigecycline for the selection of the transconjugants, respectively. The conjugation frequency was calculated as the ratio of transconjugants over recipient cells.

### 2.4 Whole-genome sequencing assembly and annotation

The genomic DNA of *K. pneumoniae* K82 was extracted using a Qiagen Minikit (Qiagen, Hilden, Germany) based on the manufacturer’s recommendations. Whole-genome sequencing was performed using both the Illumina NovaSeq platform (Illumina, San Diego, CA, United States) and the long-read PacBio RS II platform (Pacific Biosciences, Menlo Park, CA, United States). *De novo* hybrid assembly of the Illumina and PacBio reads was performed using Unicycler v0.4.8 (Wick et al., 2017). The complete genome was annotated using prokka (Thirugnanasambandam et al., 2017). Antimicrobial resistance genes (ARGs) and virulence factors were identified using AMRFinderPlus and VFanalyzer, respectively (Liu et al., 2019; Feldgarden et al., 2021). Multilocus sequence typing (MLST) and capsular typing were performed using mlst and Kaptive, respectively. Plasmid replicons were analyzed with PlasmidFinder v2.1 (Carattoli et al., 2014). Insertion sequence (IS) elements were investigated through ISFinder (Couchoud et al., 2020).

### 2.5 Analysis of plasmids with colistin or tigecycline resistance determinants of genera *Klebsiella*

In total, 7,179 complete plasmid sequences of *Klebsiella* spp. were downloaded from the National Center for Biotechnology Information (NCBI) RefSeq GenBank. The ARG profiles of these plasmids were analyzed using AMRFinderPlus, and 119 plasmids carrying complete colistin or tigecycline resistance determinants were selected for further study (Supplementary Table S1). The ability of the mobilization and conjugation of plasmid was predicted using MOB-suite (Robertson and Nash, 2018). The neighbor-joining tree was constructed on the basis of the pairwise mash distances of the plasmids using Mashtree (Robertson and Nash, 2018). The tree was midpoint rooted and visualized using Interactive Tree of Life (iTOL).

### 2.6 Data availability

The complete genome sequences of *K. pneumoniae* strain K82 were deposited in GenBank with accession numbers CP124873-CP124878.

## 3 Results

### 3.1 General characteristics of *Klebsiella pneumoniae* K82

*Klebsiella pneumoniae* K82 was isolated from the urine of a patient after bladder cancer surgery. The strain was resistant to nearly all the tested antimicrobials (Table 1), including aminoglycoside, fluoroquinolone, colistin, tetracyclines, sulfonamide, and most β-lactams (aztreonam, ceftazidime, cefepime, piperacillin/tazobactam, cefoperazone/sulbactam), but remained susceptible to carbapenems (meropenem, imipenem).

**Table 1:** Antimicrobial susceptibility of *K. pneumoniae* isolate K82, its transconjugants, and *E. coli C*600.

Antibiotics	K82 MIC (mg/L)	*E. coliC*600	*E. coli* C600/pK82-mcr-1
Ceftazidime	32 (R)	0.5	16 (32-fold)
Cefepime	≥32 (R)	≤0.125	8 (>64-fold)
Aztreonam	16 (R)	≤1	16 (>16-fold)
Imipenem	≤0.25 (S)	≤0.25	≤0.25
Meropenem	≤0.25 (S)	≤0.25	≤0.25
Piperacillin/Tazobactam	32 (R)	≤4	≤4
Cefoperazone/Sulbactam	≥64 (R)	≤8	16 (>2-fold)
Amikacin	≥64 (R)	≤2	≥64 (>32-fold)
Tobramycin	≥16 (R)	≤1	≥16 (>16-fold)
Trimethoprim-Sulfamethoxazole	160 (R)	≤20	≤20
Levofloxacin	≥8 (R)	0.5	4 (8-fold)
Minocycline	≥16 (R)	≤1	2 (2-fold)
Tigecycline	8 (R)	0.125	0.25 (2-fold)
Colistin	4 (R)	0.25	4 (16-fold)

The results of WGS showed that the complete genome of *K. pneumoniae* K82 consisted of a 5.22-Mb chromosome and five plasmids. The genomic features are summarized in Table 2. *In silico* MLST and capsular typing showed that *K. pneumoniae* K82 belonged to ST656-KL23. A total of 20 ARGs and five metal resistance operons were identified in *K. pneumoniae* K82, and most of them were located on plasmids. The colistin-resistant and tigecycline-resistant determinants were carried by pK82-*mcr-1* and pK82-*tmexCD-toprJ*, respectively.

**Table 2:** Genetic features of the KP82 genome.

Feature	Chromosome	pK82-*mcr*-*1*	pK82-*tmexCD*-*toprJ*	Plasmid3	Plasmid4	Plasmid5
Size (bp)	5,221,001	315,303	180,663	32,954	5,784	2,524
GC content (%)	57.57	47.57	51.84	34.36	45.16	44.37
CDS number	4,860	334	168	49	6	1
tRNAs number	88	0	0	0	1	0
tmRNAs number	1	0	0	0	0	0
ncRNAs number	76	1	5	0	1	1
rRNAs number	25	0	0	0	0	0
Antimicrobial resistance gene	*oqxA*, *oqxB*, *fosA*, *bla*_SHV-187_	*sul2*, *sul1*, *qnrB4*, *fosA3*, *qacE*, *aph(4)-Ib*, *aac(3)-IV*, *armA*, *bla*_CTX-M-14_, *bla*_DHA-1_, *mcr-1.1*, *mph(E)*, *msr(E)*	*aph(3″)-Ib*, *aph(6)-Id*, *tmexC1-D1:topr1*			
Metal resistance gene		*mer* operon, *ter* operon	*ars* operon, *pco* operon, *sil* operon			

### 3.2 The characteristics of plasmid pK82-*mcr-1*

**Figure 1:**
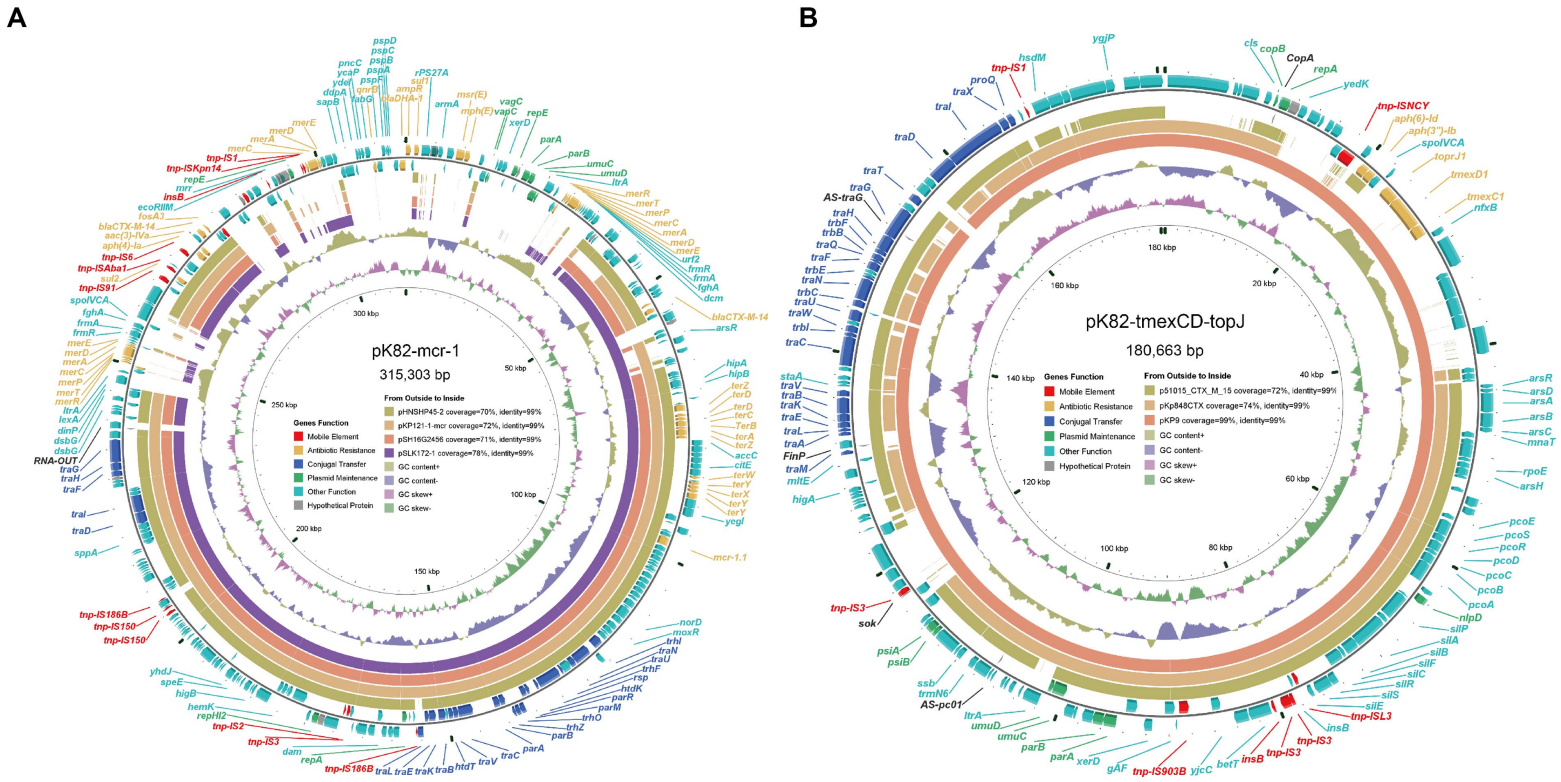
Diagram of plasmids pK82-*mcr*-*1* and pK82-*tmexCD*-*toprJ*. **(A)** The plasmid map of pK82-*mcr*-*1*; **(B)** The plasmid map of pK82-*tmexCD*-*toprJ.*

The plasmid pK82-*mcr-1* was 315,303-bp in length with an average G + C content of 47.57% (Figure 1A). A total of five replication proteins were identified on the plasmid, which belonged to the IncFIA(HI1), IncHI2, IncHI2A, IncN, and IncR family incompatibility groups. The result of the BLASTn search against the NCBI database showed that pK82-*mcr-1* shared high similarity (>70% coverage) with four *mcr-1*-carrying plasmids, the highest of which was pSLK172-1 (accession number CP017632) from the *E. coli* strain. The four plasmids belonged to the IncHI2/IncHI2A family and the backbone structure of the IncHI2/IncHI2A type plasmid, including replication, conjugative transfer system, maintenance, and stability functional regions, were conserved in these plasmids as well as pK82-*mcr-1*. The *mcr-1* gene of pK82-*mcr-1* was located between the *ISApl1* and *pap2*. The structure (*ISApl1-mcr-1-pap2*) resulted from *Tn6330* losing one copy of *ISApl1*, which was common in other *mcr-1*-carrying plasmids (Snesrud et al., 2018). A total of 12 ARGs, including *mcr-1*, three copies of mercury resistance gene clusters, and a tellurium resistance gene cluster, were identified in the MDR region of pK82-*mcr-1*. Moreover, we observed that the complete backbone sequence (from *vagC* gene to *umuD* gene) of the IncR-type plasmid was presented in the variable region of pK82-*mcr-1*. The results of the conjugation experiment showed that pK82-*mcr-1* could be successfully transferred to the recipient *E. coli* C600 at a frequency of 1.18 × 10^−4^ cells per recipient cell. The transconjugant acquired most of the antimicrobial resistance of the donor strain K82, except for piperacillin/tazobactam, cefoperazone/sulbactam, trimethoprim-sulfamethoxazole, and tetracyclines (Table 1).

### 3.3 Comparative analysis of plasmid pK82-*tmexCD-toprJ and* *tmexCD-toprJ-harboring plasmids*

pK82-*tmexCD-toprJ* was a 180,663-bp IncFIB(K)/IncFII(K) type plasmid with an average G + C content of 51.84% (Figure 1B). pK82-*tmexCD-toprJ* exhibits the highest similarity (99% coverage and 99% identity) with the *tmexCD-toprJ*-harboring plasmid pKP9 (accession number MZ690484), followed by the *tmexCD-toprJ*-negative plasmid pKp845CTX (accession number NC_024992). In addition to *tmexCD1-toprJ1*, we also found two ARGs and three metal resistance gene clusters conferring resistance to arsenic, copper, and silver (Figure 1B). Although conjugation transfer-related genes were identified on the pK82-*tmexCD-toprJ*, we could not transfer it into *E. coli* C600 or *E. coli* J53 by conjugation, which was consistent with the previous reports (Dong et al., 2022b).

To investigate the evolution of pK82-*tmexCD-toprJ*, we constructed a mash distance-based phylogenetic tree of plasmids carrying *tmexCD*-*toprJ* or its variants and aforementioned *tmexCD-toprJ*-negative plasmid pKp845CTX (Supplementary Figure S1). The results showed that pK82-tmexCD-toprJ was in the same branch, with six *tmexCD1*-*toprJ1-*harboring plasmids and pKp845CTX. All these plasmids were from *K. pneumoniae* (Supplementary Figure S1). Comparative plasmid analysis of pK82-*tmexCD-toprJ* with these seven plasmids showed that the plasmid backbone region, including maintaining stability (*parB, umuCD*), the conjugative transfer region (*tra*), and the metal resistance region (*sil, pco, ars* genes) were conserved among these plasmids (Supplementary Figure S2). The results indicate that these plasmids might have evolved from the same ancestor. In addition, we observed that plasmids MZ690482 and MZ690487 acquired more resistance genes, including *tetA* and *bla*_TEM-1_, which suggests that this type of plasmid has higher plasticity and aggravates the possibility of further spread.

### 3.4 Characteristics of plasmids with colistin or tigecycline resistance determinants of *Klebsiella* spp.

**Figure 2:**
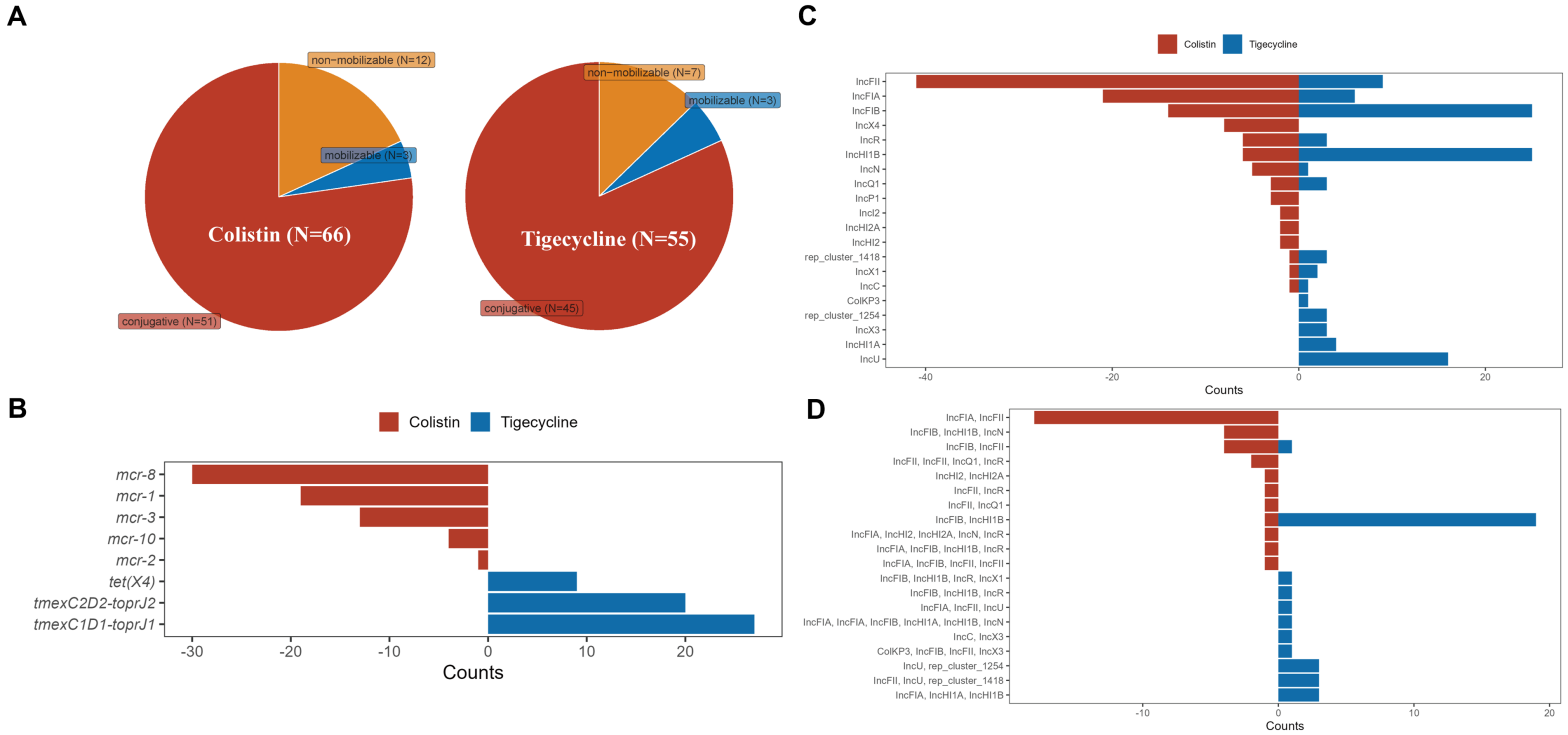
Characteristics of plasmids with colistin or tigecycline resistance determinants of *Klebsiella* spp. **(A)** The predicted transferability of the plasmids carrying CoRG or TRG; **(B)** The types of CoRG and TRG carried by the plasmids; **(C)** The Inc. group types of the plasmids carrying CoRG or TRG; **(D)** The Inc. group types of the hybrid plasmids carrying CoRG or TRG.

Totals of 119 plasmids of *Klebsiella* spp. carrying colistin or tigecycline resistance genes were obtained from GenBank for subsequent analysis together with pK82-*mcr-1* and pK82-*tmexCD-toprJ*. Among these plasmids, 67 carried CoRG, 55 carried TRG, and one plasmid, pSYCC1_tmex_287k (CP113179), carried the two resistance determinants simultaneously (Figure 2A). Most of these plasmids were predicted to be capable of conjugative transfer. The types of CoRG and TRG carried by these plasmids were five and three, respectively. The most common CoRG was *mcr-8*, while the most common TRG was *tmexCD1-toprJ1* (Figure 2B). A total of 16 and 15 Inc. groups were identified on plasmids carrying CoRG and TRG, respectively. The most common Inc. group of the former was IncFII, and the most common Inc. group of the latter was IncHI1B (Figure 2C). Notably, more than half of CoRG-harboring (52.2%, 35/67) and TRG-harboring (63.6%, 35/55) plasmids were hybrid plasmid types, in which IncFIA/IncFII and IncFIB/IncHI1B were the most common hybrid groups for CoRG-harboring and TRG-harboring plasmids, respectively (Figure 2D).

**Figure 3:**
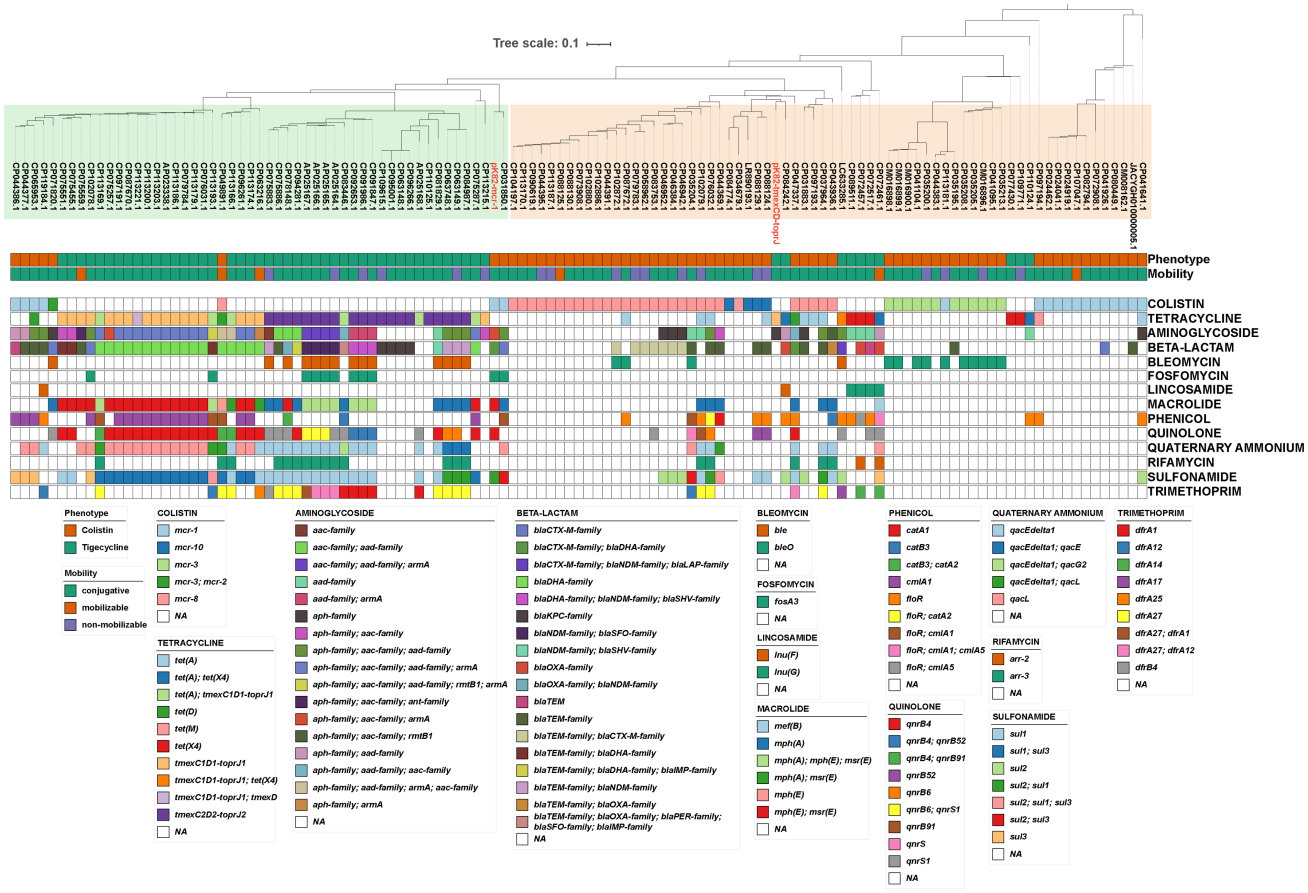
Phylogenetic analysis and antimicrobial resistance gene of plasmids carrying CoRG or TRG. Mash-based evolutionary relationships, plasmid characteristics, and antimicrobial resistance genes are shown from top to bottom, respectively. The meaning of the colored squares of each row is shown in the legends on the outside, and white means not present.

We further analyzed the antimicrobial resistance genotypes of these plasmids (Figure 3). ARGs carried by these plasmids could confer resistance to 14 classes of antimicrobials, and up to 11 classes of antimicrobial resistance could be provided by a single plasmid. Resistance to β-lactam was the most common phenotype (61.16%, 74/121) of these TRG/CoRG-harboring plasmids, some of which (16.53%, 20/121) even exhibit resistance to carbapenems. The counts of the resistance class of TRG-harboring plasmids (median = 8) were higher than that of CoRG-harboring plasmids (median = 2). The results of the mash clustering tree showed that these TRG/CoRG-harboring plasmids could be divided into two main categories (Cluster-TRG and Cluster-CoRG), which were highly in consistent with their phenotypes (Figure 3). However, we noticed that some CoRG-harboring plasmids were closely related to TRG-harboring plasmids, suggesting the possibility that a single plasmid exhibited both tigecycline and colistin resistance.

### 3.5 Co-occurrence of *mcr-1* and *tmexCD-toprJ* in one plasmid

**Figure 4:**
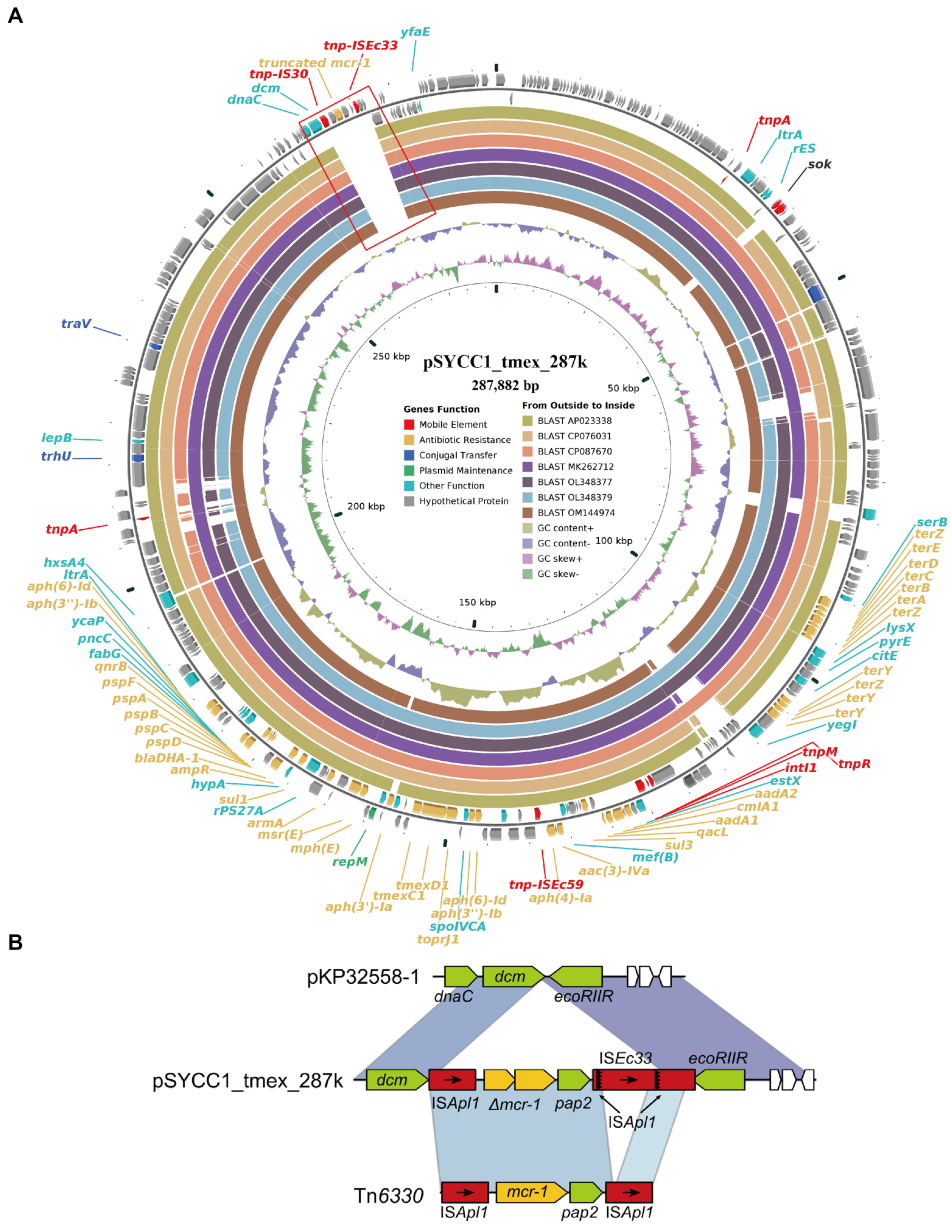
Diagram of plasmids pSYCC1_tmex_287k. **(A)** The plasmid map of pSYCC1_tmex_287k; **(B)** The genetic context of mutated mcr-1 in pSYCC1_tmex_287k.

Surprisingly, we found an *mcr-1* with a frameshift mutation encoding a truncated MCR-1 protein on the *tmexCD-toprJ*-harboring plasmid pSYCC1_tmex_287k. pSYCC1_tmex_287k was 287,882-bp in length, and its replicon belonged to the IncFIB/IncHI1B Inc. group, which was the most common Inc. group of hybrid TRG-harboring plasmid. pSYCC1_tmex_287k shared high similarity (coverage >95% and identity >99%) with seven *tmexCD-toprJ*-carrying plasmids, while intact or truncated *mcr-1* was not found on them (Figure 4A). Comparative genomic analysis revealed that pSYCC1_tmex_287k had an insertion of an *mcr-1*-harboring fragment between the *dcm* gene and *ecoRIIR* gene compared to these similar sequences (Figure 4B). The genetic context of *mcr-1* in pSYCC1_tmex_287k was almost identical to that of *Tn6330*, while *ISApl1* downstream of *pap2* was disrupted by *ISEc33* insertion. The results indicated that the plasmid pSYCC1_tmex_287k might be generated by the insertion of *mcr*-harboring Tn6330 into a *tmexCD-toprJ*-carrying plasmid of the IncFIB/IncHI1B Inc. group.

## 4 Discussion

Colistin and tigecycline are important treatment options for multidrug-resistant and pan-drug-resistant Gram-negative bacteria, especially carbapenemase-producing *K. pneumoniae* in patients with severe infection (Karakonstantis et al., 2020). The emergence of colistin and tigecycline-resistant strains presents a difficult problem for clinical treatment. In this study, we identified a multidrug-resistant *K. pneumoniae* isolate carrying the plasmid-encoded CoRG *mcr-1.1* and TRG *tmexCD1-toprJ1* simultaneously. Moreover, we characterized all plasmids with CoRG or TRG in GenBank. The findings of this study will provide a new perspective on multiple resistance mechanisms to the last-resort antimicrobials. Of note, this represents the rare report of co-occurrence of *mcr-1* and *tmexCD1-toprJ1* on a single plasmid.

*K. pneumoniae* K82 belongs to ST656, a rare clone endemic in China (Wang et al., 2012). Recently, carbapenem-resistant ST656 strains have been reported worldwide (Roberts et al., 2022). In addition to *mcr-1* and *tmexCD1-toprJ1*, K82 also carried multiple ARGs, including ESBL, as well as various metal resistance operons conferring resistance to multiple drug classes. Most of these ARGs located on IncFIA/IncHI2/IncHI2A/IncN/IncR-type plasmid pK82-*mcr-1* and IncFIB/IncFII-type plasmid pK82-*tmexCD-toprJ* were found to be associated with mobile genetic elements (MGEs), which play important roles in the resistance gene transfer (He et al., 2022). The genetic context of *mcr-1* was similar to *Tn6330*, which, apart from *ISApl1* downstream of *pap2,* was disrupted by *ISEc33* insertion. This insertion event may be of great importance in the transferability and evolution of resistance genes (Wang et al., 2017). It has been reported that *tmexCD1-toprJ1* has disseminated among *K. pneumoniae* strains from different sources, such as poultry, food markets, and patients, and is located on plasmids or chromosomes (Sun et al., 2023). Its orthologous variants, *tmexCD2-toprJ2*, *tmexCD3-toprJ3,* and *tmexCD4-toprJ4,* were reported in various species of *Enterobacteriaceae*, while the plasmid types were different from pK82-tmexCD-toprJ. The *tmexCD2-toprJ2* cluster was mainly located on IncHI1B plasmids, while the tmexCD3-toprJ3 gene cluster was located on SXT/R391 ICE (Wang Q. et al., 2021; Wang Y. et al., 2021). The *TmexCD4-toprJ4* cluster was identified on untypeable plasmids, which was closely related (92 to 99% amino acid identity) to *tmexCD1-toprJ1*, *tmexCD2-toprJ2,* and *tmexCD3-toprJ3* (Gao et al., 2022). Interestingly, recently, an ST656 *K. pneumoniae* strain carrying the *mcr-1* and *tmexCD1-toprJ1* genes was also reported, whereas the drug resistance genes were located on the IncX1 and IncR/IncN plasmids, respectively (Wang et al., 2023). These results indicate that ST656 *K. pneumoniae* is a potential high-risk clone that acquires resistance genes through various MGEs to produce resistance to almost all available antimicrobials. It is urgently vital to enhance the global surveillance.

*In silico* typing based on plasmid replicons of *Klebsiella* spp. showed that CoRG and TRG were diverse among plasmids, yet multiple genes were widely distributed across the plasmids, which is consistent with the previous reports (Algarni et al., 2022). The types of CoRG carried by plasmid replicons successively were *mcr-8*, *mcr-1*, *mcr-3*, *mcr-10,* and *mcr-2*, while the types of TRG were *tmexCD1-toprJ1*, *tmexCD2-toprJ2,* and *tet*(X4). As the above result shows, *mcr-8* and *tmexCD1-toprJ1* are the most common CoRG and TRG, respectively. The role of plasmid harboring *mcr-8* in colistin-resistant *K. pneumoniae* should be highlighted, as it indicates that the genetic context of *mcr-8* is heterogeneous and diverse (Wu et al., 2020). The crucial Inc. group was IncF, which is present in 56% of all multi-replicons, along with IncH, IncR, and IncU replicons (Douarre et al., 2020). The IncF-type plasmids are widely distributed in clinically relevant *Enterobacteriaceae* isolates (Johnson and Nolan, 2009), which is consistent with our report. These disparate plasmids contribute to the bacteria of antibiotic resistance gene dissemination among bacterial pathogens (Von Wintersdorff et al., 2016). Furthermore, there were too many ARGs carried by these plasmids, which is similar to pK82-*mcr-1*. Some horizontally transmitted accessory genes located on transposons and plasmids can be acquired by these ARGs carried by different plasmids. Worse still, the ARGs shuttle between resistant and sensitive strains, which increases resistance in different classes of antimicrobials (Acman et al., 2022). Furthermore, it poses a significant threat to global public health, especially when the genes are carried by a single plasmid among *Enterobacteriaceae* (Liu Z. et al., 2021). In our report, we noticed that some CoRG-harboring plasmids were closely related to TRG-harboring plasmids, suggesting the possibility that a single plasmid exhibits both tigecycline and colistin resistance, which presents a great threat to public health. Therefore, our study indicated that CoRG or TRG-carrying plasmids in *K. pneumoniae* strains are full of diversity, which is worth exploring more broadly.

Conjugative plasmids have facilitated the spread of antimicrobial resistance genes among clinically important pathogens (Matamoros et al., 2017). Recently, the rapid emergence of plasmid-mediated resistance genes *mcr-8* and *tmexCD1-toprJ1* confers transferable resistance to both tigecycline and colistin, which has attracted intense attention (Sun et al., 2020). Interestingly, in our study, we found an *mcr-1* with a frameshift mutation on the *tmexCD-toprJ*-harboring plasmid pSYCC1_tmex_287k (CP113179), which indicated the co-occurrence of *mcr-1* and *tmexCD1-toprJ1* on a single plasmid of the IncFIB/IncHI1B Inc. group. The coexistence of *mcr-1* and other TRGs in the same strain makes clinical treatment more challenging, and this coexistence phenomenon has been described many times (Liu Y. et al., 2021; Lu et al., 2022). However, a report showed that *mcr-1* coexisted with other TRGs on an IncHI2-type single plasmid in *E. coli* (Xu et al., 2021), which increases the challenges of controlling antibiotic resistance. It was reported that *mcr-1* and *tet*(X4)-coharboring plasmids could evolve into a plasmid with lower fitness costs, which would accelerate the transmission of *mcr-1* and TRGs globally (Lu et al., 2021). Our research extends the known diversity and mechanistic insights of CoRG and TRG-harboring plasmids in *K. pneumonia*, which might benefit the development of new antibacterial agents.

## 5 Conclusion

The present study documents a clinically isolated ST656 *K. pneumoniae* isolate co-harboring plasmid-encoded resistance gene *mcr-1* and *tmexCD1-toprJ1* from the urine specimen of a bladder cancer patient. These resistance determinants are located on distinct plasmids. In addition, the analysis of plasmids with colistin or tigecycline resistance determinants of genera *Klebsiella* reveals that the *tmexCD-toprJ*-harboring plasmid pSYCC1_tmex_287k contains a frameshift mutation *mcr-1*, which represents the first report of the coexistence of *mcr-1* and *tmexCD-toprJ* in one plasmid in China. This will inevitably accelerate the horizontal transmission of *K. pneumonia* resistance to colistin and tigecycline among *Enterobacteriaceae* species. Measures must be implemented to strengthen reasonable monitoring to avoid the spread of colistin- and tigecycline-resistant strains in China.

## Data availability statement

The datasets presented in this study can be found in online repositories. The names of the repository/repositories and accession number(s) can be found in the article/Supplementary material.

## Ethics statement

The studies involving humans were approved by Medical Ethics Committee of Ningbo Medical Center Li Huili Hospital. The studies were conducted in accordance with the local legislation and institutional requirements. The participants provided their written informed consent to participate in this study. Written informed consent was obtained from the individual(s) for the publication of any potentially identifiable images or data included in this article.

## Author contributions

QM contributed to the conception and design of the study. YZ, CQ, JY, QL, RZ, LQ, and QM participated and acquired the data. YZ conducted the experiments and drafted the manuscript. CQ analyzed and interpreted the data. All authors contributed to the article and approved the submitted version.
