# Supplementary figures and images for: Convergence of plasmid-mediated Colistin and Tigecycline resistance in Klebsiella pneumoniae
Source: Front Microbiol. 2024 Jan 3;14:1221428. doi: 10.3389/fmicb.2023.1221428 (PMC10813211; doi:10.3389/fmicb.2023.1221428)

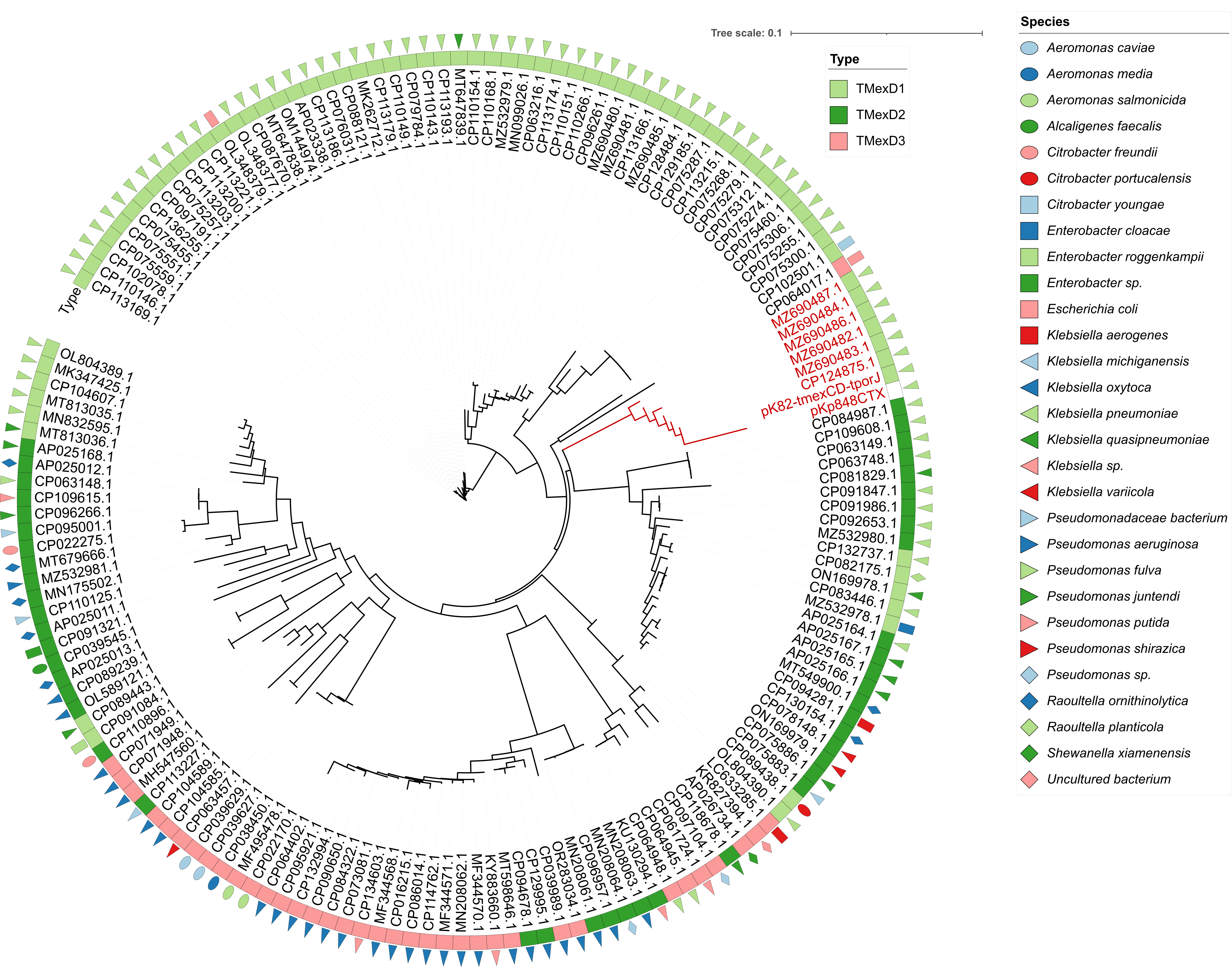

Supplement: Supplementary file 2 [file Image_1.TIF]

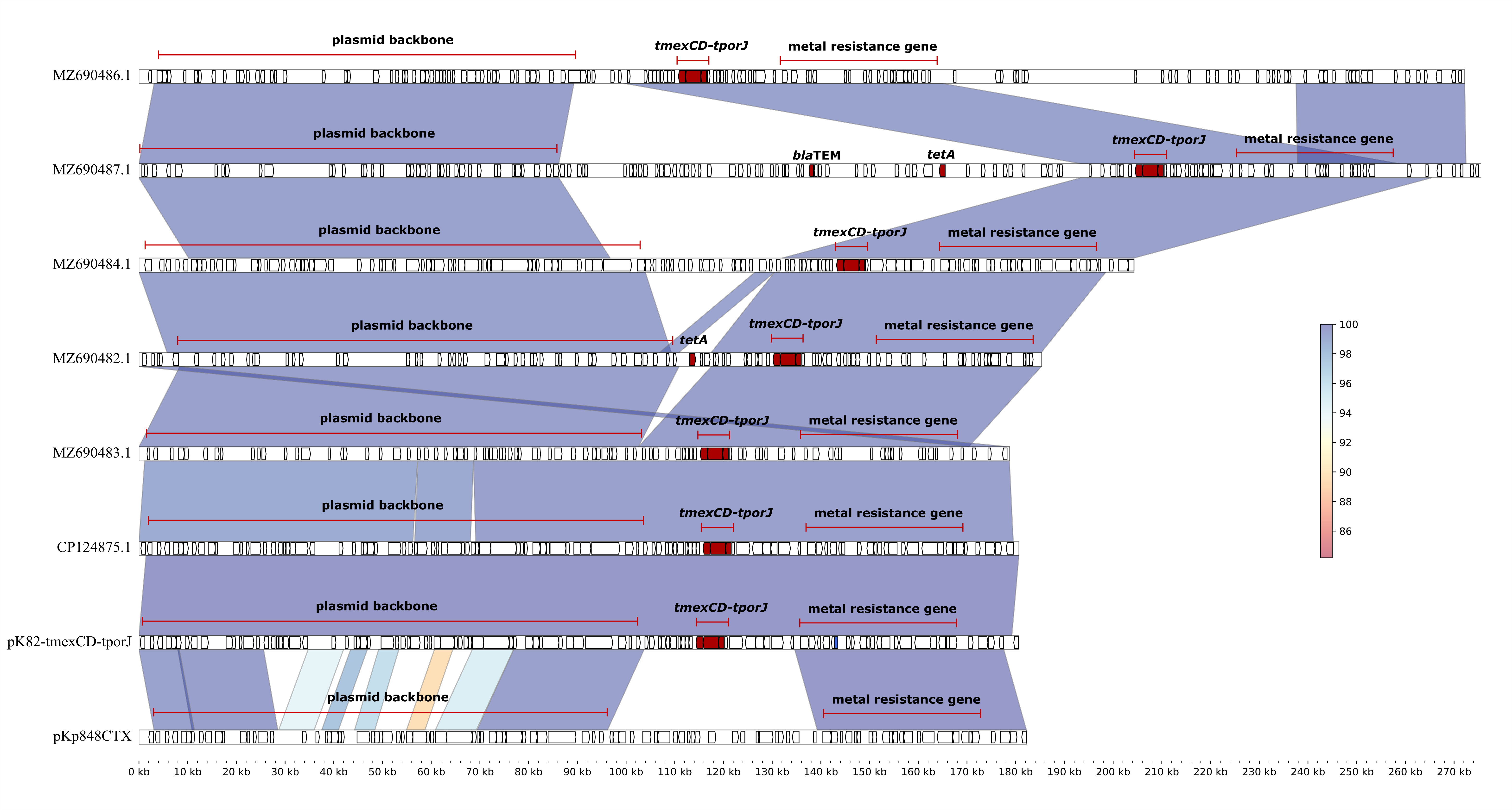

Supplement: Supplementary file 3 [file Image_2.TIF]
